# ATP-binding cassette sub-family a member1 gene mutation improves lipid metabolic abnormalities in diabetes mellitus

**DOI:** 10.1186/s12944-019-0998-3

**Published:** 2019-04-22

**Authors:** Huili Yan, Lei Cheng, Ruoshuang Jia, Huiqian Yao, Hongxia Wu, Yaqian Shen, Ying Zhang, Panpan Hao, Zhongwen Zhang

**Affiliations:** 1 Department of Endocrinology, Shandong Provincial Qianfoshan Hospital, Shandong University, School of Medicine and Life Sciences, University of Jinan-Shandong Academy of Medical Sciences, and Department of Cardiology, Shandong University Qilu Hospital, Jinan, Shandong China; 2 School of Basic Medical Sciences of Shandong University, Jinan, Shandong China; 3 Department of Surgery, Juye Coalfield Central Hospital, Heze, Shandong China; 4 Department of Endocrinology, Shandong Provincial Qianfoshan Hospital, Shandong University, Jinan, Shandong China; 5 Department of Cardiology, Shandong University Qilu Hospital, Jinan, Shandong China

**Keywords:** Diabetes mellitus, ATP-binding cassette sub-family a member1, Triglyceride, Hypertriglyceridemia

## Abstract

**Background:**

Patients with diabetes mellitus were often accompanied with hyperlipidemia. ATP-binding cassette sub-family A member1 (ABCA1) promotes the efflux of lipids and thereby mediates the metabolism of cholesterol. The aim of our study was to determine the associations of ABCA1 gene polymorphisms with the risks of diabetes mellitus and dyslipidemia in diabetic patients.

**Methods:**

We retrieved literature about the relationship between ABCA1 gene polymorphisms (C69T and R230C) and the risk of diabetes through PubMed, Web of Science, EMBASE, Wanfang Database, China National Knowledge Infrastructure (CNKI) and Cochrane database. Weighted mean difference (WMD) and odds ratio (OR) were used to compare continuous and dichotomous variables, respectively, accompanied by their 95% confidence interval (CI).

**Results:**

A total of 1746 diabetic patients and 1292 non-diabetic controls were enrolled. All subjects were Caucasians. ABCA1 R230C T allele was significantly associated with reduced the risk of diabetes (OR = 0.75, 95% CI = 0.57–0.98, *P* = 0.04). There was no association of ABCA1 C69T gene polymorphisms with the risk of diabetes. However, subgroup analyses showed that the ABCA1 C69T gene mutation significantly reduced the risk of hypertriglyceridemia in diabetic patients as compared with that in non-diabetic subjects (dominant model: WMD =0.66, 95% CI = 0.52–0.8, *P* < 0.0001; recessive model: WMD = 0.47, 95%CI = 0.11–0.83, *P* = 0.01).

**Conclusions:**

ABCA1 R230C T allele gene mutation is a protective in decreasing the risk of diabetes in Caucasians and ABCA1 C69T gene mutation markedly influences the level of lipid metabolism in diabetic patients.

**Electronic supplementary material:**

The online version of this article (10.1186/s12944-019-0998-3) contains supplementary material, which is available to authorized users.

## Background

Diabetes mellitus (DM) is one of the four majors’ non-communicable diseases identified by World Health Organization [1, 2]. In recent years, with the improvement of people’s living standard, the incidence and prevalence of diabetes are increasing year by year. Diabetes has become a serious social problem threatening people’s health and caused widespread concern in the world [1–3].

Obesity, hyperlipidemia, environmental and genetic factors, the major risk factors for the occurrence of DM, are always considered as the important research content of diabetes etiology [3, 4]. ATP-binding cassette sub-family A member 1 (ABCA1), a highly conserved transmembrane protein, is abundantly expressed on the plasma membrane and Golgi complex [5–7]. ABCA1 plays a vital role in the metabolism of cholesterol, especially high-density lipoprotein (HDL) cholesterol, through regulating the outflow of lipid to the extracellular space of peripheral cells [5–7]. Genetic studies have shown that some single nucleotide polymorphisms (SNPs) in the ABCA1 gene might cause its transcriptional activity change, affect the level of HDL cholesterol, and then lead to the susceptibility of individuals to hyperlipidemia-related diseases [5–18]. Diabetic patients were often accompanied with hyperlipidemia. Overmuch cholesterol in the cell will impairs insulin secretion in the pancreas [8–10], and possibly increases the risk of diabetes. However, the association between the ABCA1 gene polymorphisms and the risk of DM in Caucasians is still controversial [6, 7, 11–15]. Although some studies found the ABCA1 gene mutation increased the risk of diabetes [6, 12, 13], the results were negative in other people’s studies [7, 11, 15]. In addition, previous studies have failed to assessment the correlation between ABCA1 gene polymorphisms and lipid metabolism in Caucasian patients with DM. Therefore, we performed the pooled analysis to evaluate the relationships between the ABCA1 gene polymorphisms and the risks of diabetes and dyslipidemia in diabetic patients.

## Methods

### Literature retrieval strategy

We retrieved articles about the relationship between ABCA1 gene polymorphisms and DM through the PubMed database, Web of Science, Wanfang Database, EMBASE, China National Knowledge Infrastructure (CNKI) and Cochrane database, using the terms ‘ABCA1’, ‘ATP-binding cassette sub-family A member 1’, ‘ATP-binding cassette’ in combination with ‘type 2 diabetes mellitus’ or ‘type 1 diabetes mellitus’ or‘diabetes mellitus’ or ‘DM’ or ‘hyperlipidemia’ or ‘cholesterol’ or ‘triglyceride’. We had no restrictions on the languages of the articles, the publication types of the articles or whether the articles had been published. The pooled analysis was carried out based on the Preferred Reporting Items for Reviews and Meta-Analyses (PRISMA) statement.

### Study selection criteria

Eligible articles met the following criteria: (1) Case–control studies: diabetic patients as the case group, non-diabetic subjects in the control group; (2) Prospective cohort studies; (3) Studies related to the ABCA1 gene polymorphisms and the risk of DM; (4) If more than one study were carried out by the same research group, only the trial with the most complete data is included in our pooled analysis. Exclusion criteria included: (1) Besides diabetes mellitus and dyslipidemia, patients have other diseases also related to the ABCA1 gene polymorphisms; (2) Non case–control studies; (3) Animal or in vitro research; (4) Insufficient in formation concerning evaluation rates.

### Statistical analysis

A dichotomous variable was used to the evaluate the relationship between the mutation of the ABCA1 gene and the risk of DM. A continuous variable was used to the evaluate the association between single nucleotide polymorphism (SNP) of the ABCA1 gene and the serum lipid level in patients with or without DM. Dichotomous variable were analyzed by using odds ratios (OR) and 95% confidence interval (CI). The weighted mean difference (WMD) and 95% CI were used to compare the continuous variable. The Hardy–Weinberg equilibrium was tested by the χ^2^-test. Heterogeneity was assessed by Cochrane’s Q test and the I^2^ statistic. In pooled-analysis, heterogeneity is suspected if *P*-value < 0.10 in Cochrane’s Q-test, moreover, 25% ≤ I^2^ < 50% and I^2^ ≥ 50% are considered as evidences of modest and high heterogeneity, respectively. When the heterogeneity I^2^ is less than 50%, the fixed effect model was merged, otherwise, the random effect model was used. The correlation between ABCA1 gene mutation and DM was analyzed by the following methods: ABCA1-C69T C/T (dominant model: CT + TT vs CC, recessive model: TT vs CC + CT) and ABCA1- R230C C/T (dominant model: CT + TT vs CC, recessive model: TT vs CC + CT). The risk frequency of T allele (both C69T and R230C) was also calculated in our meta analysis. The subtypes of diabetes mellitus (type 1 diabetes or type 2 diabetes), body mass index (BMI) and the serum lipid level (triglycerides (TG), low density lipoprotein (LDL) cholesterol, total cholesterol and high-density lipoprotein (HDL) cholesterol) were selected for stratified analyses. The Rosenthal’s fail-safe number (N_fs_) and funnel plot were used to test the publication bias. The formula N_fs_0.05 = (ΣZ/1.64)2 - k (k is the number of articles included in this research). The significance of Nfs was set at 0.05 for each meta comparison. If the calculated N_fs_0.05 value was smaller than the number of studies, indicating the research might have publication bias. Values of *P* < 0.05 were considered as significant differences. All analyses were carried out using Review Manager 5.2 (Cochrane Collaboration, Oxford, UK).

## Results

In total, 467 potentially relevant studies retrieved by the initial search. A preliminary screening of 22 trials might be related to our research. Seventeen trials were identified after 5 repetitive trials were removed. After carefully reading the full text of the 17 trials, 6 suitable trials were included. A total of two types of ABCA1 gene polymorphism (rs1800977 and rs9282541) were screened. All the subjects in both groups were Caucasians. For ABCA1 rs1800977 (C69T) polymorphism, 3 case-control studies that included 647 diabetic patients and 595 non-diabetic subjects were entrolled. For ABCA1 rs9282541 (R230C) polymorphism, 3 case-control studies that included 1099 diabetic patients and 697 non-diabetic subjects. All the patients in the case groups were diagnosed as type 2 diabetes. The main characteristics of the included studies are listed in Table 1.

**Table 1:** Main characteristics of the included studies in the pooled analysis

First author	Year	Ethnicity	Subjects, n cases/controls	Mean age, y cases/controls	FBG in cases/controls (mmol/l)	CHO in cases/controls (mmol/l)	TG in cases/controls (mmol/l)	HDL in cases/controls (mmol/l)	LDL in cases/controls (mmol/l)	Test for HWE X^2^ p
Three studies for the ABCA1-C69T polymorphism										
Khalid K Alharbi	2013	Caucasian	376/380	50.6 ± 10.3/46.0 ± 7.6	9.40 ± 1.5/8.69 ± 1.82	5.63 ± 1.26/5.05 ± 0.97	2.23 ± 1.65/1.61 ± 0.86	0.93 ± 0.75/0.64 ± 0.23	3.79 ± 1.07/3.68 ± 0.85	2.64
Polin Haghvirdizadeh	2015	Caucasian	164/165	62.1 ± 9.5/54.9 ± 11.7	8.66 ± 3.50/5.45 ± 1.31	4.38 ± 1.14/4.43 ± 1.17	1.76 ± 1.20/1.27 ± 0.76	1.26 ± 0.48/1.21 ± 0.43	2.35 ± 0.90/2.62 ± 0.95	21.61
H. Arzu Ergen	2012	Caucasian	107/50	NA/NA	9.85 ± 3.76/5.29 ± 1.41	5.49 ± 1.50/3.94 ± 0.71	1.96 ± 1.10/1.23 ± 0.39	0.97 ± 0.24/0.98 ± 0.36	3.67 ± 1.38/2.46 ± 0.67	0.59
Three studies for the ABCA1-R230C polymorphism										
Polin Haghvirdizadeh	2015	Caucasian	173/165	62.1 ± 9.5/54.9 ± 11.7	8.66 ± 3.50/5.45 ± 1.31	4.38 ± 1.14/4.43 ± 1.17	1.76 ± 1.20/1.27 ± 0.76	1.26 ± 0.48/1.21 ± 0.43	2.35 ± 0.90/2.62 ± 0.95	4.23
Desmond D. Campbel	2012	Caucasian	800/367	63.0/54.6	NA/NA	NA/NA	NA/NA	NA/NA	NA/NA	0.01
J.C. Lara-Riegos	2015	Caucasian	126/165	51.6/42.1	10.3 ± 5.1/5.3 ± 0.61	4.7 ± 1.0/4.6 ± 0.97	2.2 (1.6–3.2)/1.8 (1.3–2.5)	1.0 ± 0.24/1.0 ± 0.29	2.8 ± 0.84/2.8 ± 0.79	21.61

*FBG* Fasting blood glucose, *CHO* Cholesterol, *TG* Triglyceride, *HDL* High density lipoprotein, *LDL* Low density lipoprotein, *NA* Not available, *HWE* Hardy Weinberg equilibrium

There were no association of ABCA1-C69T gene polymorphisms with the risk of diabetic (dominant model: OR = 0.66, 95% CI = 0.24–1.83, *P* = 0.42; recessive model: OR = 0.56, 95% CI = 0.17–1.91, *P* = 0.36, Additional file 1: Figure S1 and Additional file 2: Figure S2). Considering the high heterogeneity in the meta-analysis (dominant model: I^2^ = 94%, recessive model: I^2^ = 91%), the lipid composition, age, BMI, and blood pressure were selected for subgroup analyses. The results showed that the lipid composition and age were correlated with the heterogeneity. Compared with the control group, the ABCA1 C69T gene mutation significantly reduced the risk of hypertriglyceridemia in diabetic patients (dominant model: WMD = 0.66, 95% CI = 0.52–0.8, *P* < 0.0001, I^2^ = 60%; recessive model: WMD = 0.47, 95%CI = 0.11–0.83, *P* = 0.01, I^2^ = 32%). The average age of diabetic patients was significantly higher than that of control group (dominant model: WMD = 7.49, 95% CI = 4.59–10.39, *P* < 0.0001, I^2^ = 0%; recessive model: WMD = 7.67, 95%CI = 3.17–12.17, *P* = 0.0008, I^2^ = 0%). No significant differences were found in the total cholesterol, HDL cholesterol, LDL cholesterol, BMI, and blood pressure between the diabetic patients and the control subjects (data no shown) (Table 2).

**Table 2:** Association of ABCA1 polymorphism and the risk of diabetes mellitus with a dominant model

Category	N	Subjects, cases/controls	Heterogeneity	OR (95% CI)	Z test
			P_h_ I^2^ (%)		
Three studies for the ABCA1-C69T polymorphism					
Overall	3	647/595	< 0.00 94	0.66 (0.24–1.83)	Z = 0.80; P_Z_ = 0.42
Adjustment by the lipid composition					
CHO	3	317/383	0.002 84	0.40 (−0.18–0.99)	Z = 1.36; P_Z_ = 0.17
TG	3	317/383	0.08 60	0.54 (0.28–0.80)	Z = 4.00; P_Z_ < 0.0001
LDL	3	317/383	0.006 80	0.06(−0.37–0.48)	Z = 0.27; P_Z_ = 0.79
HDL	3	317/383	0.004 82	0.09(−0.07–0.25)	Z = 1.09; P_Z_ = 0.27
Adjustment by blood pressure					
SBP	2	141/109	0.13 57	6.60 (−1.64–14.85)	Z = 1.57; P_Z_ = 0.12
DSP	2	141/109	< 0.00 94	1.36(−1.33–4.05)	Z = 0.99; P_Z_ = 0.32
Three studies for the ABCA1-R230C polymorphism					
C allele	3	2198/1394	0.18 42	1.34 (1.02–1.76)†	Z = 2.11; P_Z_ = 0.04
T allele	3	2198/1394	0.18 42	0.75(0.57–0.98)†	Z = 2.11; P_Z_ = 0.04

*CHO* Cholesterol, *TG* Triglyceride, *HDL* High density lipoprotein, *LDL* Low density lipoprotein, *SBP* systolic blood pressure, *DBP* diastolic blood pressure, *N* number of invealed studies, *Ph P* values for heterogeneity of Q test; † Fixed-effects model. P_Z_ < 0.05, indicate significant association

For the ABCA1- R230C gene mutation (C → T), because of the study published by J.C. Lara-Riegos was lack of datas on the number of CC/CT/TT genotypes in the control group, therefore, the risk frequency of T or C allele was calculated to evaluate the relationship between the ABCA1 R230C gene mutation and the risk of DM. The results showed that the ABCA1 R230C T allele was significantly decreased in the diabetic patients than that in the non-diabetic subjects (OR = 0.75, 95% CI = 0.57–0.98, *P* = 0.04, Additional file 3: Figure S3). A significant positive correlation was found between the ABCA1 R230C C allele gene and DM risk (OR = 1.34, 95% CI = 1.02–1.76, *P* = 0.04, Additional file 4: Figure S4). These results suggested that the ABCA1 R230C T allele gene mutation is a protective gene for decreasing the risk of diabetes in Caucasians (Table 2).

### Assessment of publication bias

The N_fs0.05_ was 174.25. It’s large enough to illustrate the stability of the results. The possibility that the conclusion of this meta-analysis was to overthrow is very small.

## Discussion

Gemiological studies of SNPs can be used to explore the correlation between the candidate genes and the risk of diseases. In the current study, we analyzed the correlations between ABCA1 gene polymorphisms and patients with diabetes mellitus using a pooled analysis to obtain a powerful conclusion. ABCA1 C69T gene mutation significantly reduced the risk of hypertriglyceridemia in diabetic patients than that in the non-diabetic subjects. There was a positive evidence that ABCA1 R230C T allele gene has an influence on the risk of DM. To the best of our knowledge, this is the first pooled analyses in Caucasian population examining the correlation between the ABCA1 gene polymorphisms and the risk of diabetes.

Lipid toxicity has recently been shown to play a pivotal role in the pathogenesis of diabetes [19]. Previous studies have found that patients with diabetes often accompanied by high levels of triglyceride and free fatty acid. High levels of free fatty acid contribute to impaired glucose phosphorylation, glucose transport, glucose oxidation and glycogen synthesis similar to the defects observed in diabetes [20, 21]. ABCA1 plays a vital role in cholesterol reverse transport and HDL production [14]. Mutations in the ABCA1 gene is correlated with HDL- cholesterol deficiency syndrome, which is characterized by near-zero plasma levels of HDL cholesterol and cholesterol esters are accumulated in macrophages [22]. In this pooled analysis, we found that there was significant differences between ABCA1 genotypes and subjects for R230C. ABCA1 R230C C allele had a significant association with higher risk of DM. ABCA1 R230C/C230C genotypes might confers susceptibility to diabetes through insulin secretion and adipocyte function [14–16]. Previous studies have shown that ABCA1 is expressed in the islet beta cells, and the expression of ABCA1 plays a critical role in maintaining normal insulin secretion. ABCA1 deficiency led to cholesterol accumulation in the plasma membrane of islet beta cells, and selective loss of ABCA1 in islet β cells led to age-related progressive impairment in glucose tolerance even in heterozygous mice with ABCA1 gene knockout, suggesting that excessive cholesterol may inhibit insulin secretion [16, 17].

ABCA1 plays a crucial role in adipocyte cholesterol homeostasis. Previous studies found that ABCA1 R230C/C230C genotypes were associated with obesity. Although the R230C mutation has not yet been functionally detected, it is likely to lead to a decrease in transporter activity. Decreased ABCA1 transporter activity led to adipocyte enlargement, which is a feature of obesity. Obesity causes insulin resistance and is implicated as one of the main determinants of diabetes [14, 17]. Moreover, some studies have shown that ABCA1 R230C/C230C genotypes appears to be specific for the Amerindian and Amerindian-derived populations. For example, the C230 allele had a frequency of 0.109 in Mestizos, whereas, ABCA1 R230C/C230C genotypes have not been found in Chinese, African or European populations [18]. Further trials are still necessary to confirm the association between ABCA1 R230C/C230C and DM.

ABCA1 C69T gene mutation significantly reduced the risk of hypertriglyceridemia in diabetic patients than that in non-diabetic subjects.. The results exhibited important information about the ABCA1 genes polymorphisms and the risk of metabolic-related diseases. Our study only focused on diabetes and did not evaluate the correlation of the ABCA1 gene polymorphisms and other disease, such as obesity, cerebral apoplexy and coronary artery disease [22–25]. In addition, the ABCA1 gene is just one of the hosts of genetic risk factors for lipid metabolic abnormalities in the diabetes population. Other factors, such as glucose, insulin, cytokines, ghrelin, leptin, and other indices of insulin resistance may also participate in the abnormal lipid metabolism in diabetic patients. Therefore, more high-quality studies in the further are required to demonstrate its true effects.

Some limitations in our study should be considered. First, all included studies were published in English or Chinese, which might result in potential language bias. Second, in the course of the study, various confounding factors (such as smoking, drinking and gender) might have different effects on the authenticity of the results. We carefully reviewed the included studies, but these included studies did not disclose more details. More studies are needed to eliminate confounding factors in the future. Third, the sample sizes in these studies were relatively small, and more studies are still necessary to confirm our results. Moreover, all included studies involved type 2 but not type 1 diabetes.

## Conclusions

Notwithstanding these limitations, our pooled analyses provided preliminary evidence of the correlation between ABCA1 gene polymorphism and diabetes in Caucasians, and supported the hypothesis that the ABCA1 gene polymorphism is a protective factor for decreasing the risk of lipid metabolic abnormalities in diabetic patients.

## Additional files

Additional file 1: **Figure S1:** ABCA1-C69T polymorphism and diabetes mellitus in a dominant model. (TIF 514 kb)

Additional file 2: **Figure S2:** ABCA1-C69T polymorphism and diabetes mellitus in a recessive model. (TIF 33 kb)

Additional file 3: **Figure S3:** ABCA1-R230C T allele mutation and diabetes mellitus. (TIF 33 kb)

Additional file 4: **Figure S4:** ABCA1-R230C C allele mutation and diabetes mellitus. (TIF 33 kb)

## Abbreviations

ABCA1: ATP-binding cassette sub-family A member1
DBP: diastolic blood pressure
DM: diabetes mellitus
FBG: fasting blood glucose
HDL: high density lipoprotein
HWE: hardy Weinberg equilibrium
LDL: low density lipoprotein
SBP: systolic blood pressure
T2DM: type 2 diabetes mellitus
TG: triglyceride
