# Supplementary figures and images for: ATP-binding cassette sub-family a member1 gene mutation improves lipid metabolic abnormalities in diabetes mellitus
Source: Lipids Health Dis. 2019 Apr 22;18:103. doi: 10.1186/s12944-019-0998-3 (PMC6477720; doi:10.1186/s12944-019-0998-3)

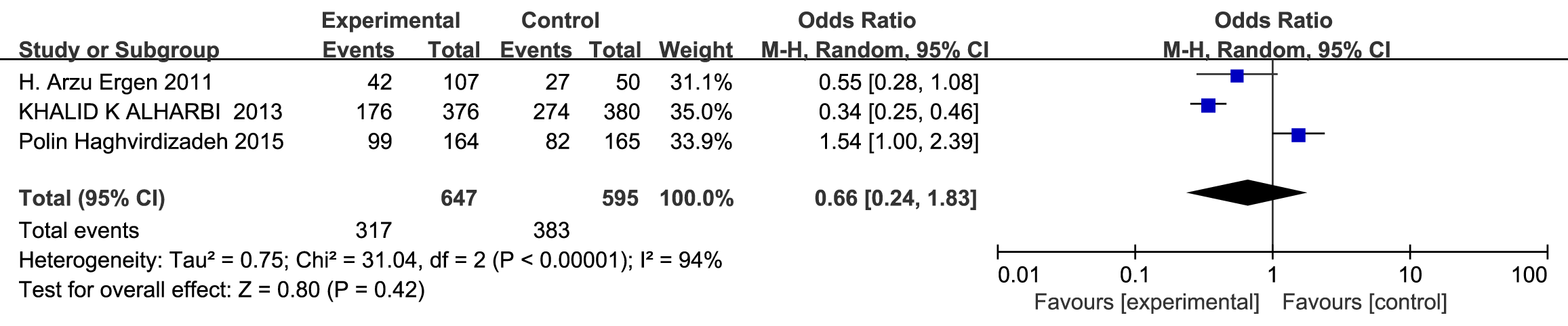

Supplement: Supplementary file 1 — Figure S1: ABCA1-C69T polymorphism and diabetes mellitus in a dominant model. (TIF 514 kb) [file 12944_2019_998_MOESM1_ESM.tif]
